# Short bowel syndrome as an unusual complication of strangulated congenital diaphragmatic hernia: Case report

**DOI:** 10.1016/j.ijscr.2020.06.103

**Published:** 2020-07-10

**Authors:** Reema AlSadhan, Abdulaziz K Alaraifi, Magdy Abdulatif

**Affiliations:** a King Faisal Specialist Hospital and Research Center, Riyadh, Saudi Arabia; b Department of Surgery, King Abdulaziz Medical City, Ministry of National Guard Health Affairs, Riyadh, Saudi Arabia; c Department of Pediatric Surgery, Maternity and Children Hospital, Al Ahsa, Saudi Arabia

**Keywords:** Congenital diaphragmatic hernia, Short bowel syndrome, Strangulated congenital diaphragmatic hernia, Recurrent congenital diaphragmatic hernia

## Abstract

**Introduction:**

Strangulation is a rare complication of congenital diaphragmatic hernia (CDH). There are few cases in the literature describing strangulated CDH. However, none of them was a recurrence of a previously repaired defect nor resulted in short bowel syndrome.

**Presentation of case:**

We report an unusual case of newborn presenting with CDH that was repaired shortly after birth but developed recurrence with strangulation few months after, requiring a massive resection, resulting in short bowel syndrome (SBS). It was managed via total parenteral nutrition feeding for three months until the patient was able to tolerate orally.

**Discussion:**

There were one familiar case in the reported literature by Woolley of an infant developing bowel infraction as a result of CDH where he later developed short bowel syndrome.

**Conclusion:**

Short bowel syndrome is an unlikely outcome of strangulated CDH. Early repair of the defect should be promoted to avoid the devastating consequences of an CDH similar to the ones encountered in our case. High index of suspicion should be kept during the follow-up of patients with CDH post repair to detect early signs of recurrences.

## Highlights

• Congenital diaphragmatic hernia is a rare cause of neonatal bowel obstruction.
• Congenital diaphragmatic hernia is associated with many long-term complications involving multiple systems.
• Only few cases in the literature has reported a strangulated congenital diaphragmatic hernia and even a fewer ones required bowel resection.
• Short bowel syndrome is an unlikely outcome of strangulated congenital diaphragmatic hernia, requiring feeding by total parenteral nutrition.

## 1 Introduction

Congenital Diaphragmatic Hernia (CDH) is a congenital anomaly seen in 1 of every 3000 live births with the left side of the diaphragm being affected in 85% of the cases [1,2]. Despite the improvement in the survival rate of newborns with CDH, the morbidity rate remains high with several reported complications such as pulmonary hypoplasia, gastric volvulus, intestinal malrotation, congenital heart defects, and renal hypertrophy [[3], [4], [5], [6], [7], [8]]. Recurrence of CDH occurs in 10% of patients with both prenatal patient-related factors and postnatal treatment-related factors playing a role in the occurrence of such event [9].

Strangulation is frequently seen in most types of hernias but it is rare in the diaphragmatic ones [10]. There are few cases in the literature describing strangulated CDH [11,12]. However, none of them was a recurrence of a previously repaired defect nor resulted in short bowel syndrome [13,14]. This paper is written with the objective of reporting a new case of a strangulated recurrent CDH that required massive intestinal resection resulting in short bowel syndrome. The work has been reported in line with the SCARE criteria [15].

## 2 Case presentation

A 3625 g infant was born to a 45-year-old female (Gravida 10, Para 9). The patient was diagnosed prenatally in another facility with CDH. The mother came to the hospital after the onset of labor and the rupture of membranes. Baby was born via cesarean section. The infant was observed to be in respiratory distress, flaccid, cyanosed, hypotensive and he had delayed crying. On physical examination, the chest was inspected to be bulging and the abdomen was scaphoid. Chest auscultation reveled a decrease air entry bilaterally with the left side being more affected than the right side where the heart sounds were more prominent. Apgar scores were three in 1 min, seven in 5 min, and seven in 10 min. Patient was shifted to neonatal intensive care unit (NICU) and was kept on mechanical ventilation.

**Fig. 1:**
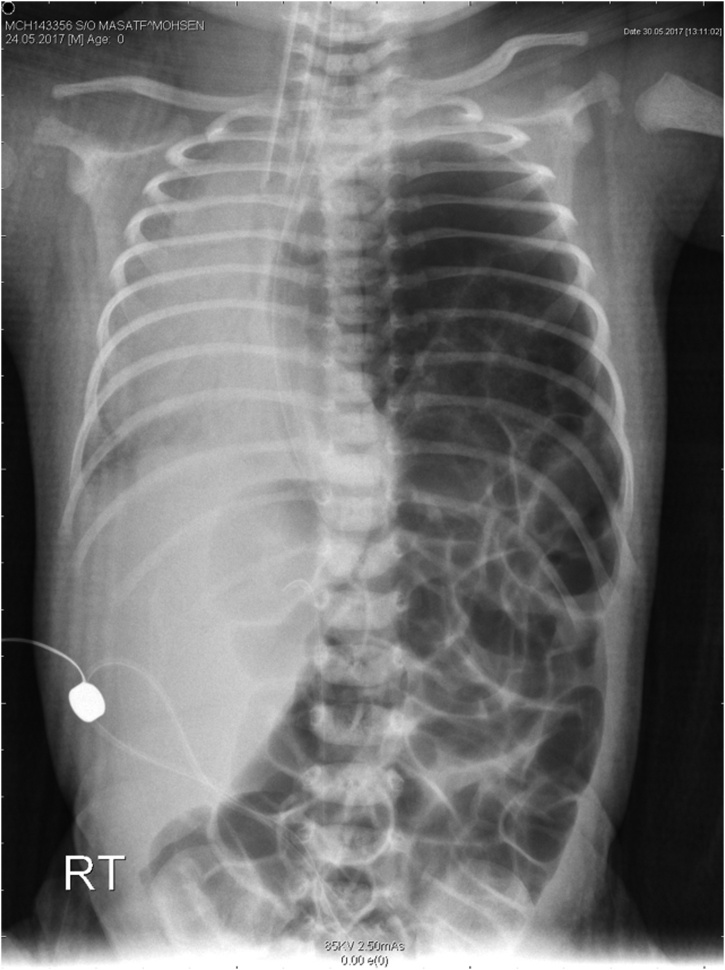
Chest x-ray showing expansion and increasing lucency of the left hemithorax with bowel loops seen within the left hemithorax and mediastinal shift to the right side.

Chest x-ray was obtained that displayed left CDH with bowel loops on the left side of the chest [Fig. 1]. Echocardiography showed tricuspid regurgitation (TR), patent ductus arteriosus (PDA), and pulmonary hypertension.

On the eighth day, CDH repair was done via a subcostal incision. The hernia’s sac was eviscerated and excised. Dissection of the posterior and anterior leaf was done followed by closure with simple interrupted sutures. Examination of the whole small bowel revealed no ischemia therefore no resection was done. Testing the closure was declared to be successful after the lungs were inflated and no bubbles were observed. Drain was inserted in the thoracic cavity with suction and the patient was transferred to the NICU. Postoperative period was uneventful as the patient was extubated on the ninth postoperative day.

**Fig. 2:**
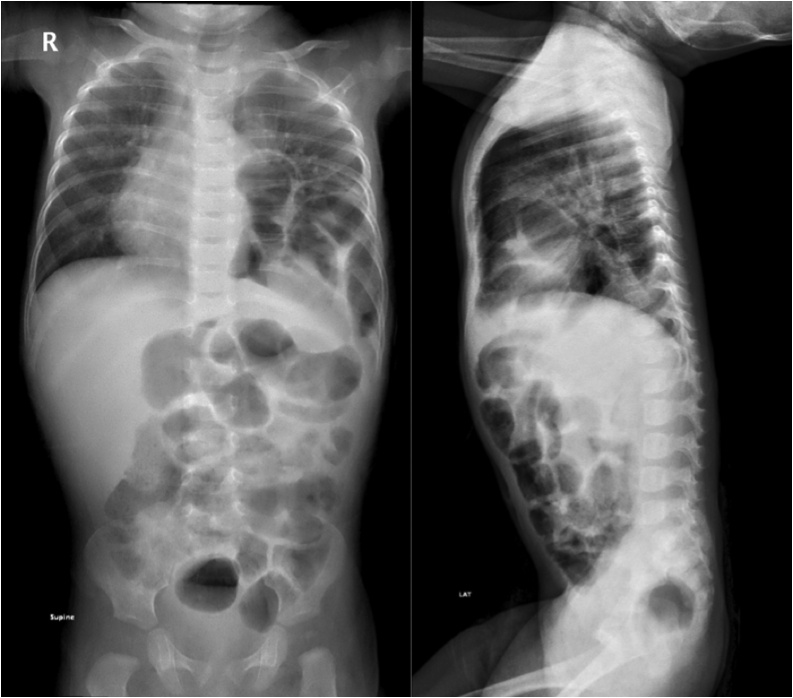
Anteroposterior and lateral chest x-ray showing bowel segments in the left hemithorax demonstrating recurrence of CDH.

**Fig. 3:**
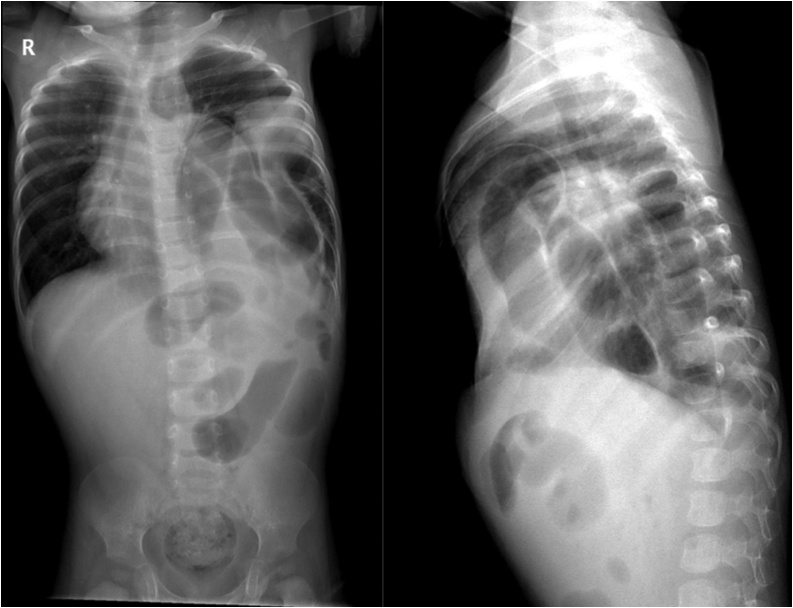
Anteroposterior and lateral chest x-ray showing strangulation CDH.

After the patient was discharged, he had multiple emergency department visits and admissions due to multiple events of respiratory tract infections and respiratory distress. Recurrence was detected at 242 days of age during follow-up and an elective repair for the hernia was scheduled [Fig. 2]. Unfortunately, before the scheduled elective repair of the hernia, the patient presented with intestinal obstruction due to strangulation of the CDH [Fig. 3] that required an emergency operation thirteen weeks after the detection of the recurrence. Through transabdominal subcostal incision, the hernia’s defect was visualized and widened in order to reduce the bowel the chest cavity. Large mesenteric defect measuring 20 cm was seen. In addition, patchy gangrenous segments of the bowel were observed requiring massive resection of the middle segment located between 90 cm from the duodenal-jejunal flexure and 25 cm from the ileocecal valve measuring about 90 cm of gangrenous bowel. Approximately 10 cm of the gangrenous small bowel was left for a second look operation. The hernia’s defect was closed and mesh placed.

Following the operation, the patient had a cardiac arrest for five minutes and received cardiopulmonary resuscitation (CPR). The former event resulted in hypoxic ischemic encephalopathy (HIE) that was confirmed via electroencephalography (EEG) and magnetic resonance imaging (MRI). Four days later, the patient underwent a second look operation where 6 cm additional gangrenous bowel segment was resected. Two stomas were created and they were closed eight days after.

The patient developed several complications following the CDH repair operation. First, he developed disseminated intravascular coagulation (DIC). Second, disseminated candida tropicalis and septic shock with multiple organ dysfunction. Third, due to the extensive bowel resection, the patient developed short bowel syndrome (SBS) requiring a prolonged total parenteral nutrition (TPN). Nine weeks after the bowel resection, he was switched to oral feeding but he couldn’t tolerate it as he developed vomiting and had to be fed via TPN once again that was gradually weaned off to NGT feeding. Fourth, he developed gastroesophageal reflex disease (GERD) grade II and had poor sucking due to HIE that formed an obstacle to oral feeds. Regardless, the patient was discharged a month later after 103 days of hospitalization on oral feeds [Table 1].

**Table 1:** Timeline of events.

Day	Event
**1**	Patient was born with respiratory distress and chest x-ray confirmed the diagnosis of CDH
**3**	Echocardiogram demonstrated TR, VSD, and pulmonary hypertension
**8**	First repair of CDH was done. The hernia sac was existed and the defect was closed
**11**	Accidently extubating the patient due to nursing error and his condition deteriorated
**18**	Signs of improvement are seen and oral feeds are started
**33**	Shifted out of NICU
**242**	CDH recurrence was detected via chest x-ray taken for pneumonia
**339**	Strangulation of the recurrent CDH developed requiring emergency operation with hernia’s defect closure and massive bowel resection. The patient had cardiac arrest following the operation that led to the development of HIE
**342**	Second look operation was done with additional bowel resection and creation of two stomas resulting in SBS
**345**	Disseminated candida tropicalis infection with septic shock and multiple organ dysfunction
**363**	Distal loop gram demonstrated bowel patency
**398**	Stoma closure
**400**	Patient is passing meconium
**407**	Patient developed vomiting after being switched to oral feeds therefore he was put once again on TPN
**442**	Patient discharged on oral feeds

## 3 Discussion

CDH is a rare cause of neonatal bowel obstruction [10,16]. Only few cases in the literature has reported a strangulated CDH and even a fewer ones required bowel resection [[10], [11], [12], [13],[16], [17], [18], [19]]. Our patient underwent a massive bowel resection leaving 105 cm of the small bowel. As a result of that, he developed SBS requiring a prolonged feeding via TPN for 3 months, which is longer than the 7 days average duration of TPN feeding after CDH repair [20]. In 1977, Woolley reported the only case in the literature reporting similar outcome that was a complicated case of CDH requiring extensive bowel resection necessitating TPN feeding for 9 months [17]. Early recurrence following repair is estimated to be 2.3% [21]. Several factors have been highlighted to be associated with recurrence of CDH in the literature such as ECMO, thoracoscopic repair, and patch repair [21,22]. None of these factors has been present in our patient. Despite the absence of these factors in our patient, the diaphragmatic hernia had recurred. The timing of the repair of the recurrence was delayed in our case for social issues. Literature shows that primary repair is preferred in neonates because it avoids the infectious and mechanical complications that comes along with usage of prosthesis [23].

With the improvement in the survival rate comes the downside of the long-term sequelae CDH survivors are at risk of developing that require long-term follow up. These morbidities include numerous systems: pulmonary – gastrointestinal – neurological – auditory – musculoskeletal [24]. Our patient experienced several complications through the course of primary repair up to the time of the discharge following the second repair. The most devastating complication our patient encountered was developing septic shock due to the bowel strangulation leading to DIC and cardiac arrest. The latter has led to HHE producing neurological impairment in our patient.

Our patient presented with recurrent respiratory infections after the repair was done, which is a complication that can occur in 37% post CDH repair [25]. This can be explained by the decrease in the lung perfusion contributing to the increase frequency of respiratory infections [25]. GERD was diagnosed in our patient following the second repair as it’s a common consequence following CDH repair occurring in 44% of cases [25].

Cardiopulmonary defect is known to be associated with CDH as a result of in-utero pressure of the bowel on thoracic structures causing developmental arrest [26]. Our patient was born with multiple cardiac defects, namely TR and PDA, alongside pulmonary hypertension and pulmonary hypoplasia. The timing of the primary repair of the defect was deferred to eight days after birth in order to stabilize the patient’s cardiopulmonary status. Delaying the primary repair till reduction of the pulmonary pressure showed a higher survival rate reaching 79–92% [13].

In conclusion, SBS is an unlikely outcome of strangulated CDH. Early repair of the defect should be promoted to avoid the devastating consequences of an CDH similar to the ones encountered in our case. High index of suspicion should be kept during the follow-up of patients with CDH post repair to detect early signs of recurrences.

## Declaration of Competing Interest

None.

## Funding

None.

## Ethical approval

Case reports do not require ethical approval in children and maternity hospital – Alahsa.

## Consent

Consent has been obtained from the patient’s mother on behalf of the patient for the publication of this case report.

## Author contribution

Reema AlSadhan: Study concept and design – data collection – writing the paper.

Abdulaziz K Al Araifi: Data collection – writing the paper.

Magdy Abdulatif: Study concept and design – reviewing of the paper.

## Registration of research studies

Not applicable.

## Guarantor

Reema AlSadhan.

## Provenance and peer review

Not commissioned, externally peer-reviewed.
